# Supplementary figures and images for: Targeting n-myristoyltransferases promotes a pan-Mammarenavirus inhibition through the degradation of the Z matrix protein
Source: PLoS Pathog. 2024 Dec 3;20(12):e1012715. doi: 10.1371/journal.ppat.1012715 (PMC11658702; doi:10.1371/journal.ppat.1012715)

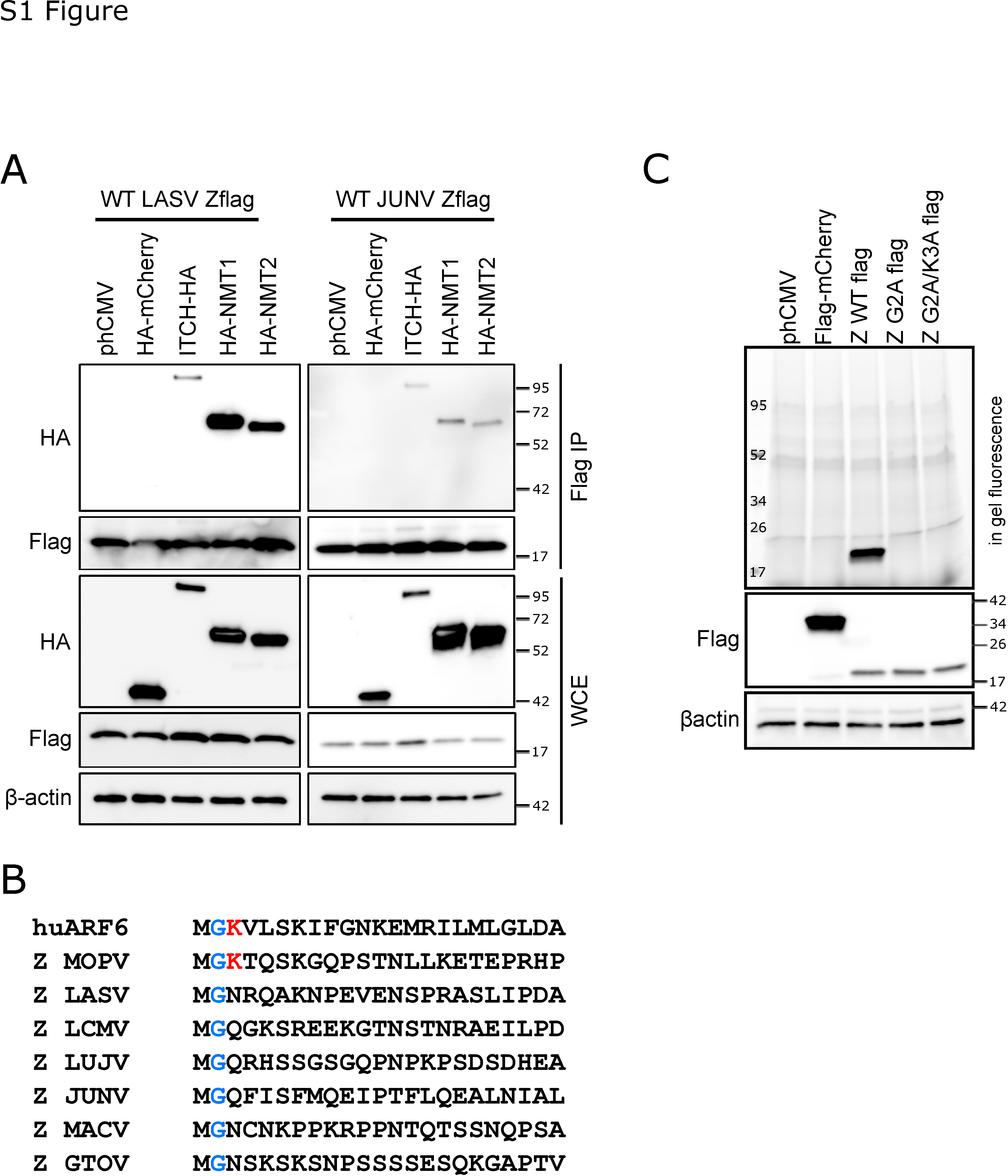

Supplement: S1 Fig — A) The Z matrix protein of LASV and JUNV interact with NMT1 and NMT2. HEK293T cells were cotransfected for 24 h with the indicated plasmids before cell lysis and Flag immunoprecipitation. WCE and IP products were analyzed by Western Blot for the presence of Flag (Z matrix protein of LASV and JUNV), HA (NMT1, NMT2 and ITCH) and β-actin. B) Sequence alignment of the ARF6 N-terminal protein, a known K3 myristoylated human protein and the Z matrix N-terminal of MOPV, LASV (Josiah), LCMV (WE), LUJV, JUNV (Espindola), MACV (Carvallo) and GTOV (INH-95551). The G2 residue is highlighted in blue and the K3 residue in red. C) Evidence for the absence of K3 myristoylation of the MOPV Z matrix protein. HEK293T cells were transfected with control plasmids or plasmids expressing the WT, G2A or G2A/K3A mutants of the MOPV Z matrix protein and treated with AzC12 (10μM). WCE were submitted to Alk-AF647 bio-orthogonal ligation or Western Blot for the detection of Flag (the Z matrix protein) and β-actin. Results in A and C) are representative of two independent experiments. (TIF) [file ppat.1012715.s001.tif]

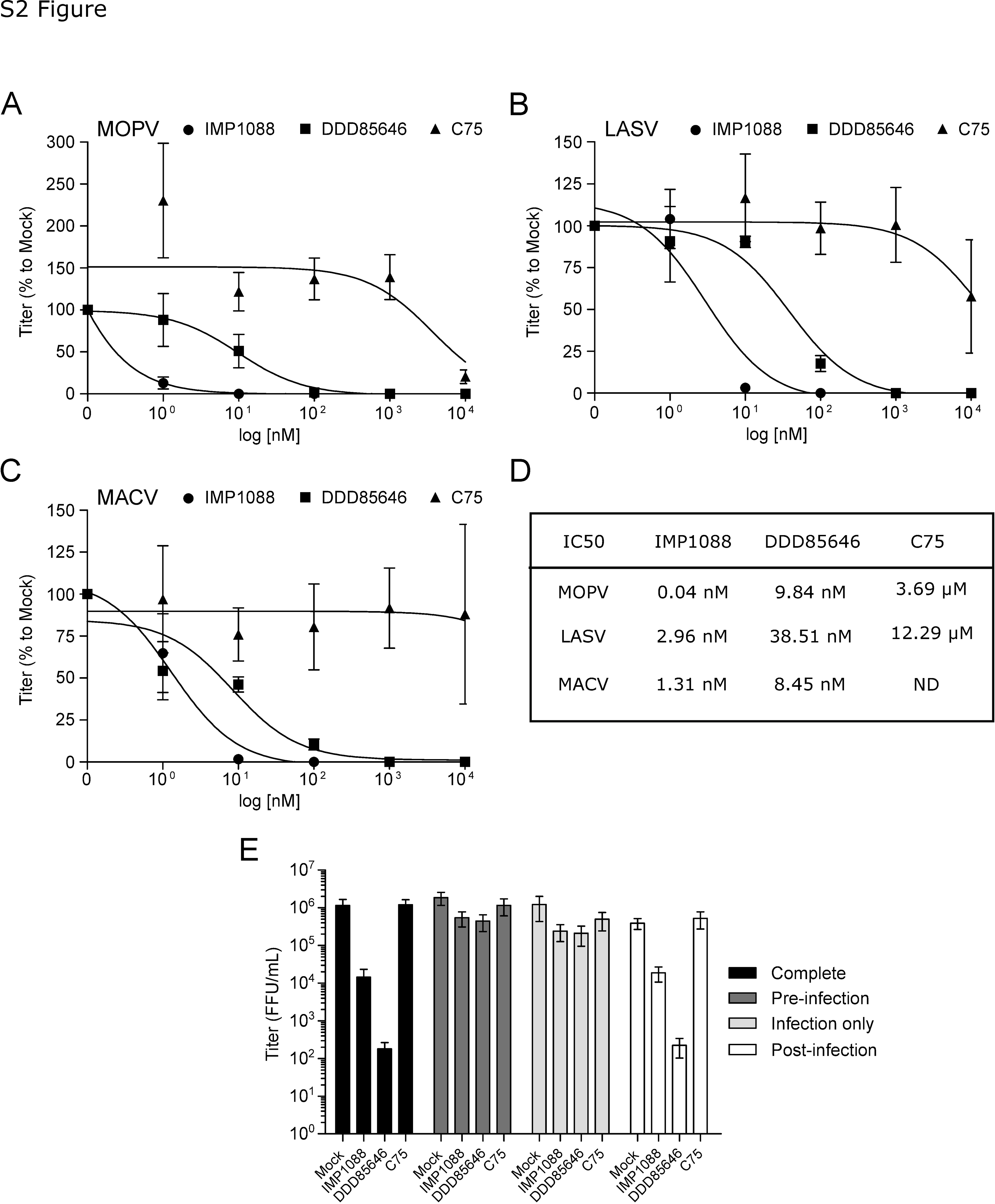

Supplement: S2 Fig — A-C) Curve fitted analyses of the results from Fig 4A–4C. Results for MOPV (A), LASV (B) and MACV (C) were obtained after normalization to the Mock condition and expressed as percentage of infectious titers to Mock. D) IC50 for IMP1088, DDD85646 and C75 for MOPV, LASV and MACV in A459 cells. E) A549 cells were treated or not with IMP1088 (20nM), DDD85646 or C75 (500nM) for 1 h before infection (Pre-infection), during infection with rMOPV WT at MOI 0.01 for 1 h (Infection only), after infection (for 48 h, Post-infection) or throughout the experiment (Complete). Cell culture supernatant were collected and titrated. The results of four independent experiments are expressed as mean +/- SEM to the viral titer (FFU/mL). (TIF) [file ppat.1012715.s002.tif]

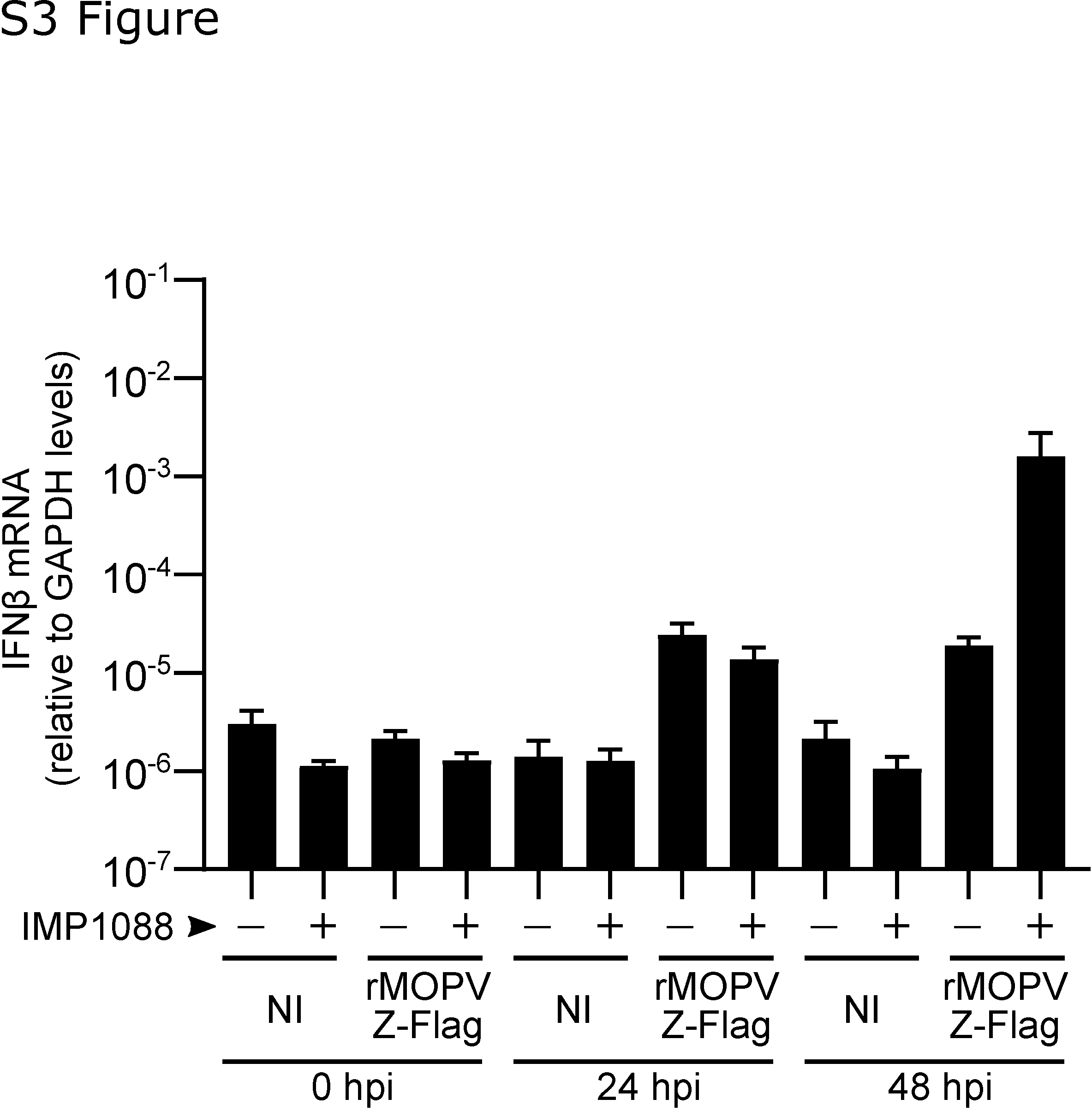

Supplement: S3 Fig — A549 cells were treated or not with IMP1088 (50nM) and infected or not with rMOPV Z-Flag at MOI 0.01 for 48 h. Total RNA and WCE were collected at 0 h, 24 h and 48 h post infection. mRNA levels for β-IFN were quantified and standardized to mRNA GAPDH levels. Results are mean +/- SEM of three independent experiments. (TIF) [file ppat.1012715.s003.tif]

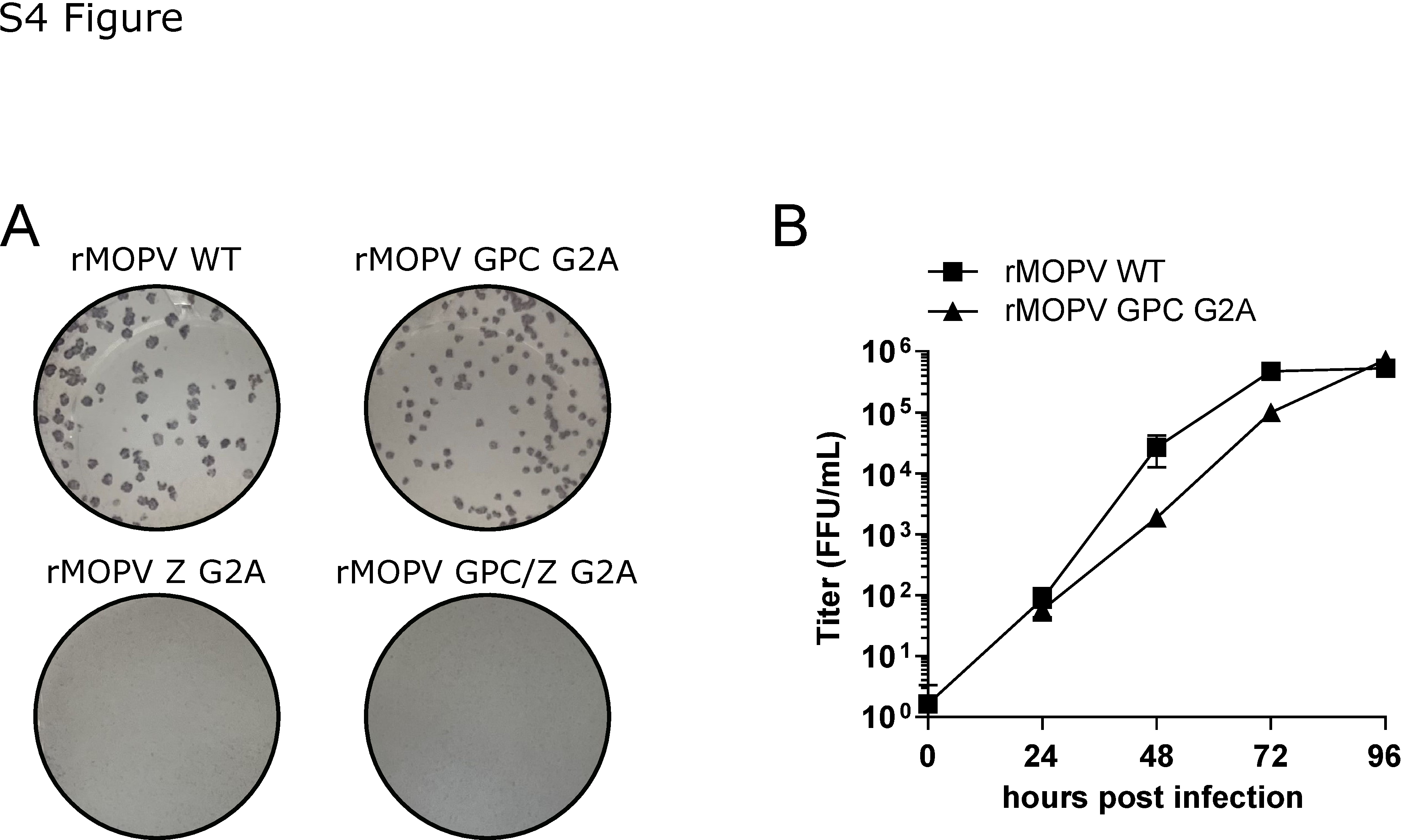

Supplement: S4 Fig — (A) Results of the rescue and plaque phenotypes of WT, GPC G2A, Z G2A and GPC/Z G2A rMOPV. (B) Comparison of the kinetics’ growth of WT and GPC G2A rMPOV in Vero E6 cells. Cells were infected at MOI 0.01 and supernatants were collected for 96 h and titrated. Results from technical triplicates are represented as mean +/- SEM to the viral titer (FFU/mL). (TIF) [file ppat.1012715.s004.tif]
