# Supplementary material for: Targeting n-myristoyltransferases promotes a pan-Mammarenavirus inhibition through the degradation of the Z matrix protein
Source: PLoS Pathog. 2024 Dec 3;20(12):e1012715. doi: 10.1371/journal.ppat.1012715 (PMC11658702; doi:10.1371/journal.ppat.1012715)

Non numerical data for Carnec et al.

TARGETING N-MYRISTOYLTRANSFERASES PROMOTES A PAN-*MAMMARENAVIRUS* INHIBITION THROUGH THE DEGRADATION OF THE Z MATRIX PROTEIN

Figure 1A

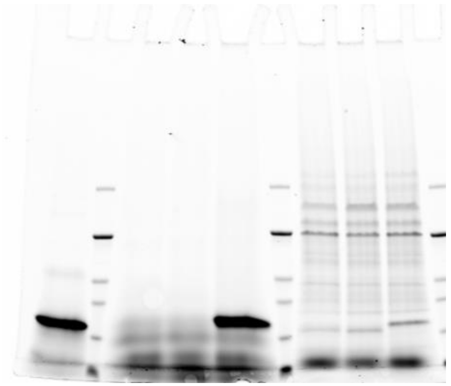

Figure 1B

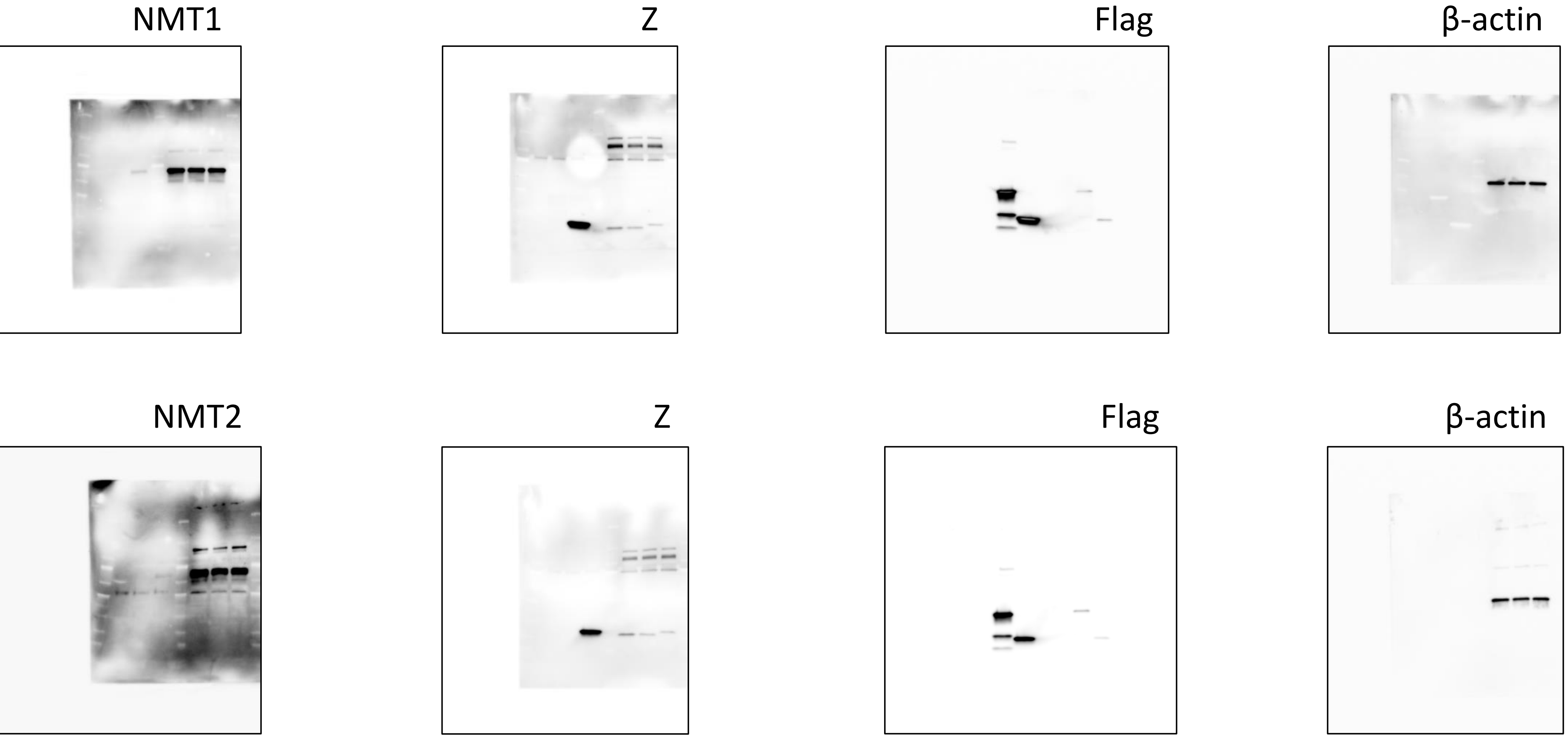

Figure 1C

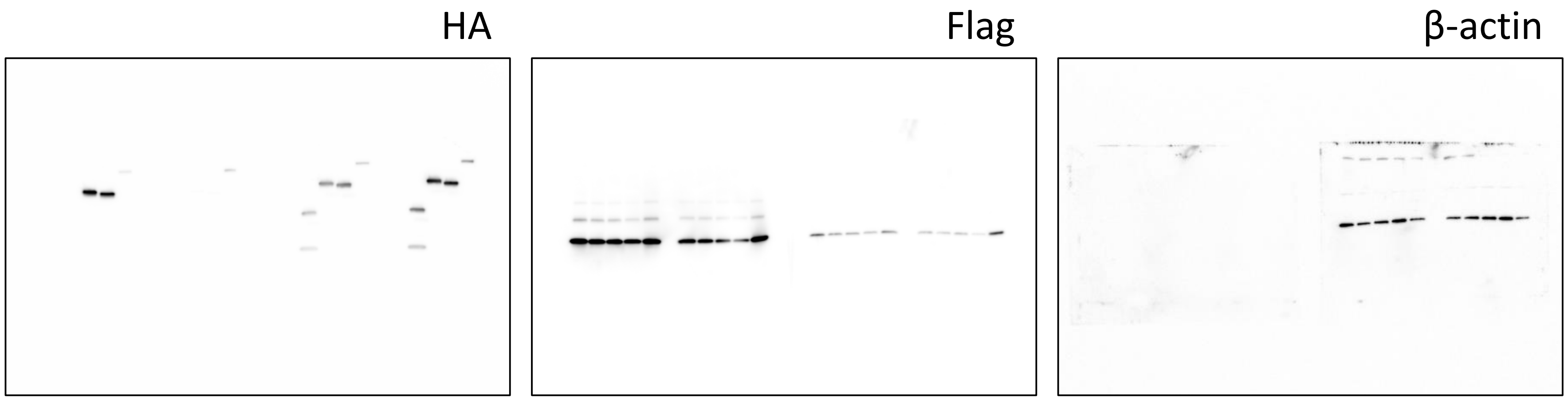

Figure 1D

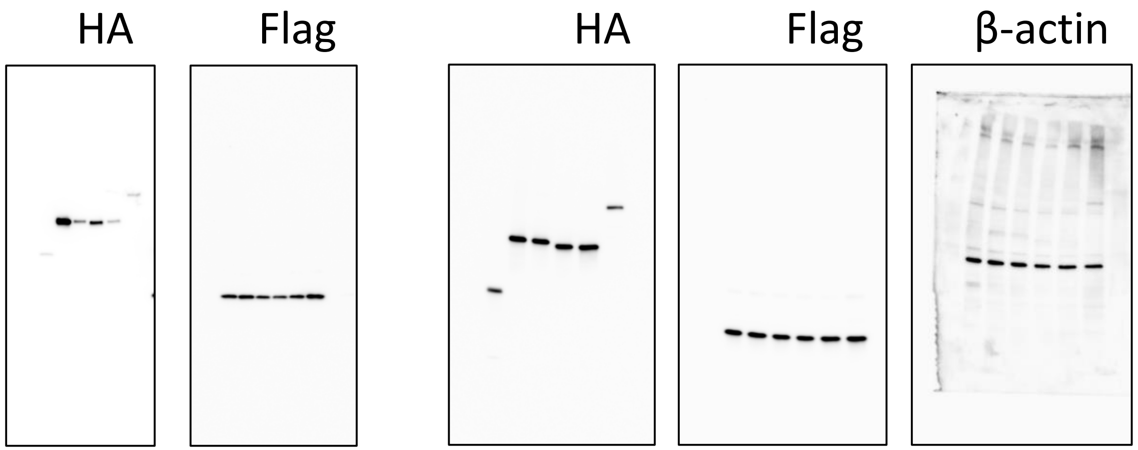

Figure 2A

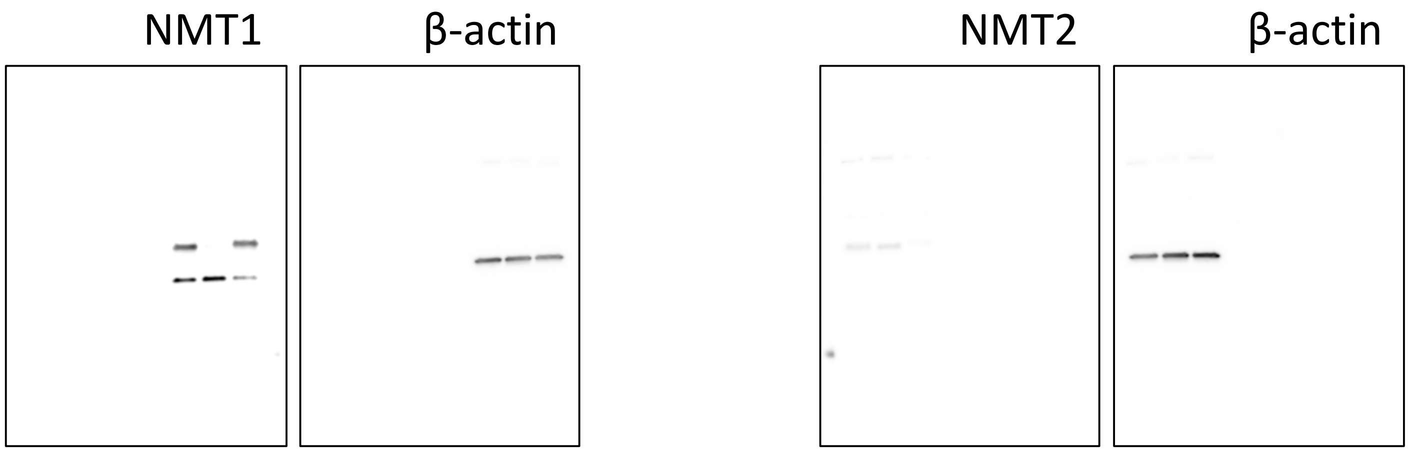

Figure 3A

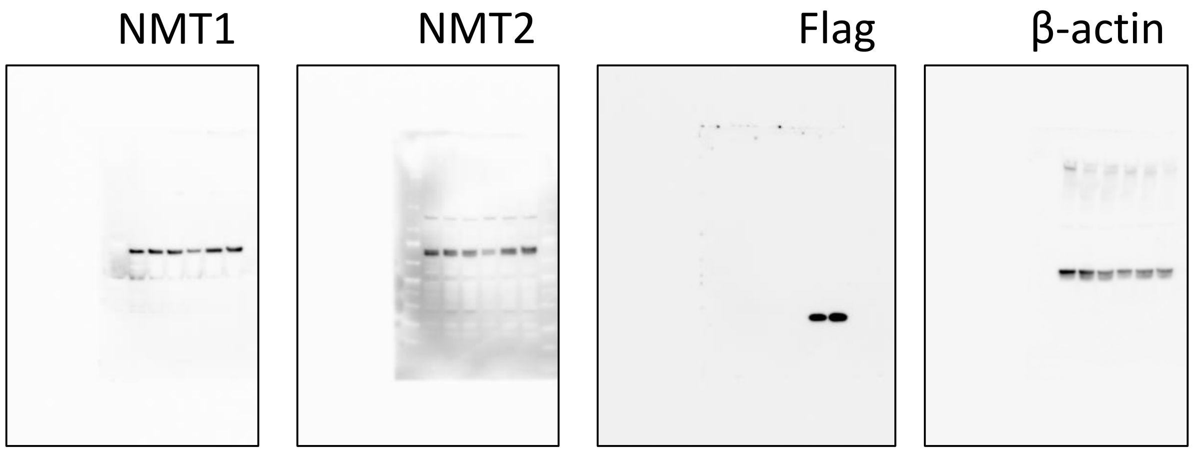

Figure 3B

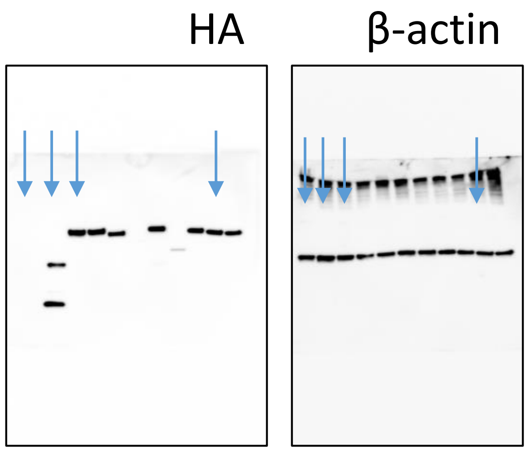

Figure 3C

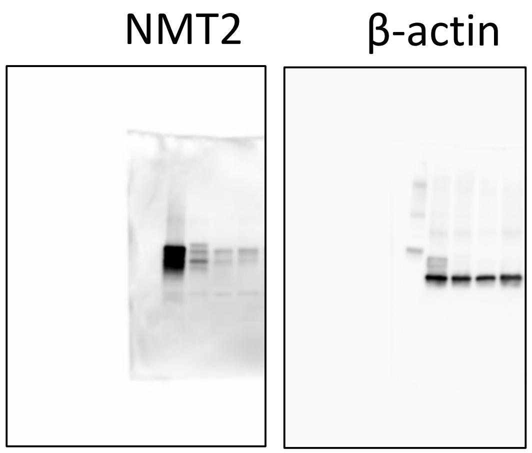

Figure 5A

In gel fluorescence and silver staining

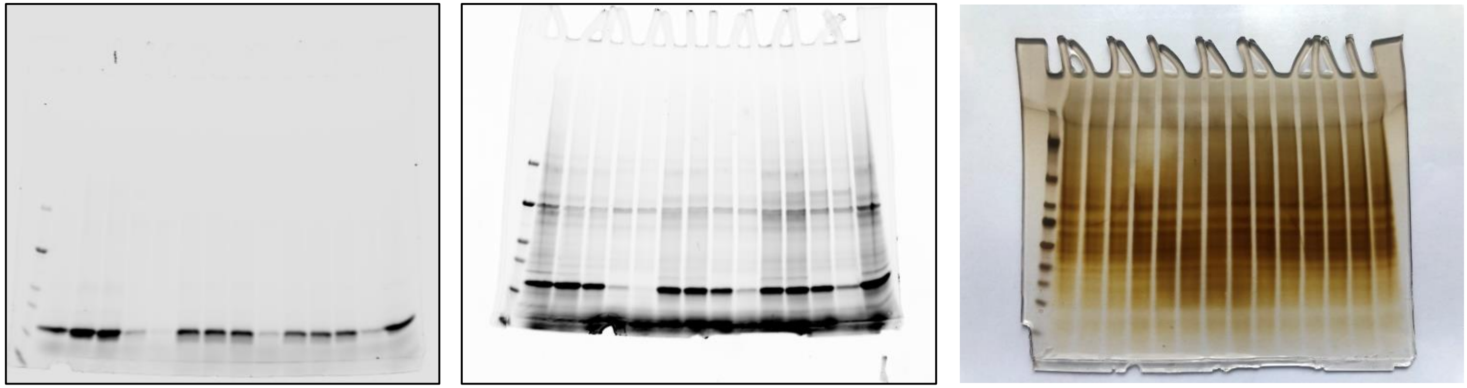

Figure 5B

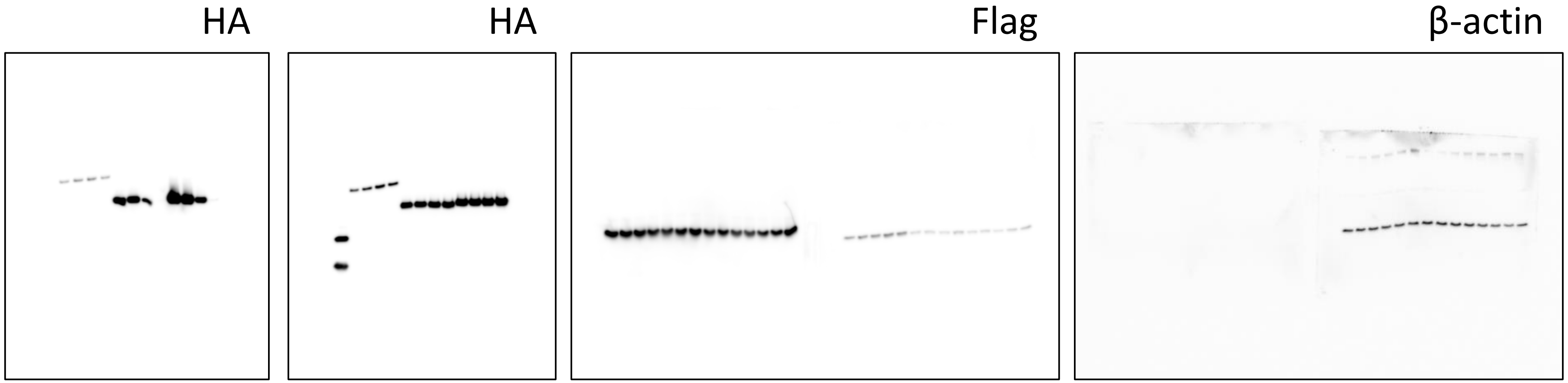

Figure 6A

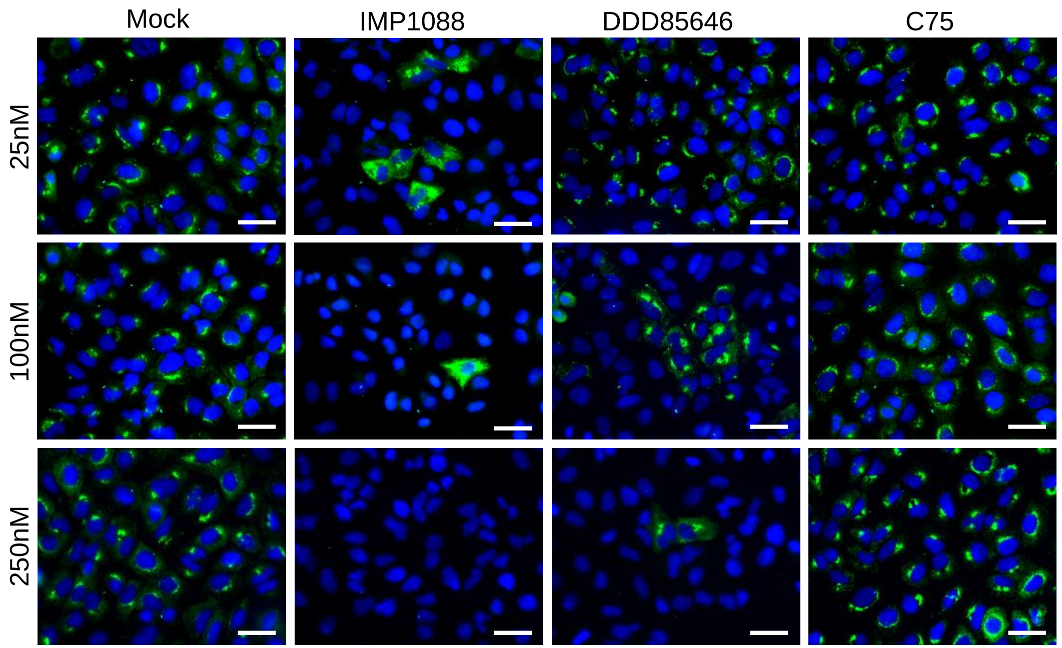

Figure 6C

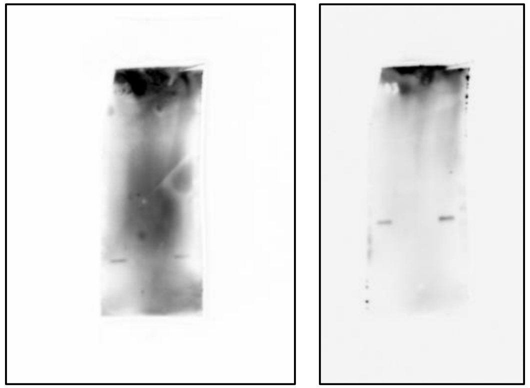

Figure 6D

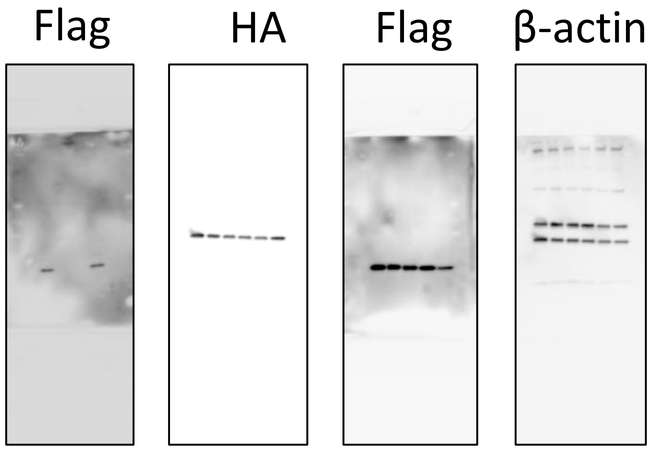

Figure 7A

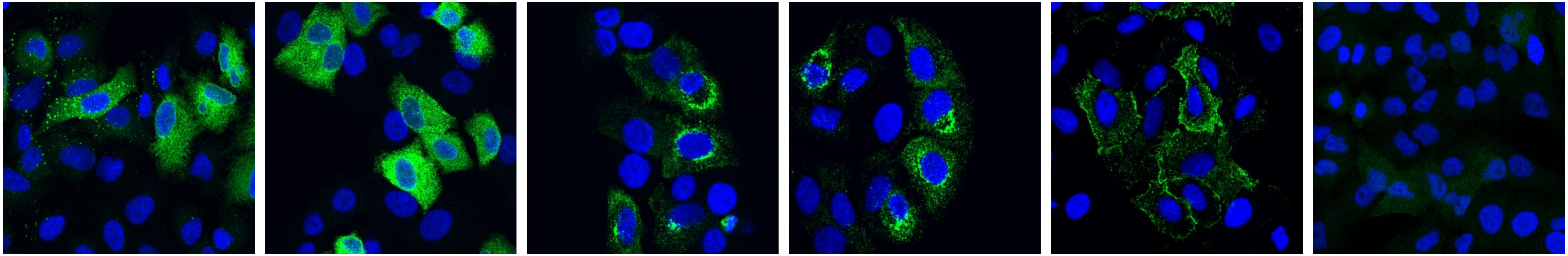

Figure 7D

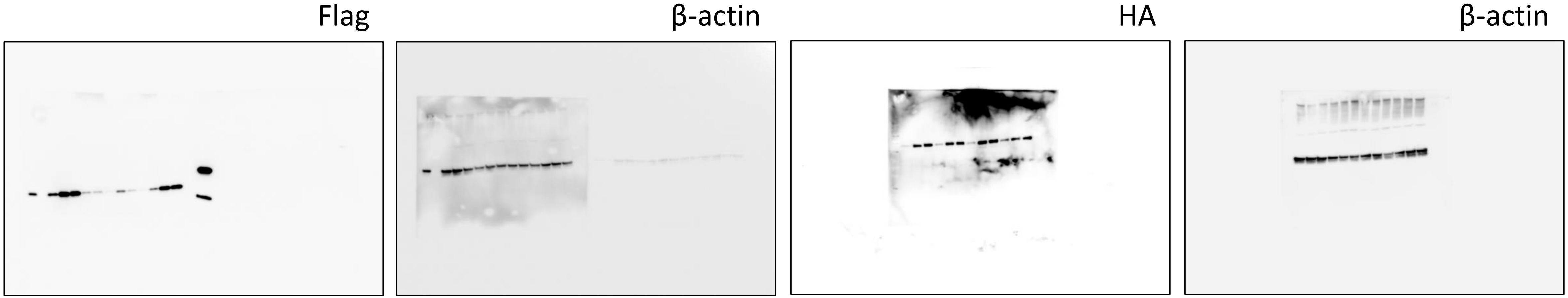

Figure 8A

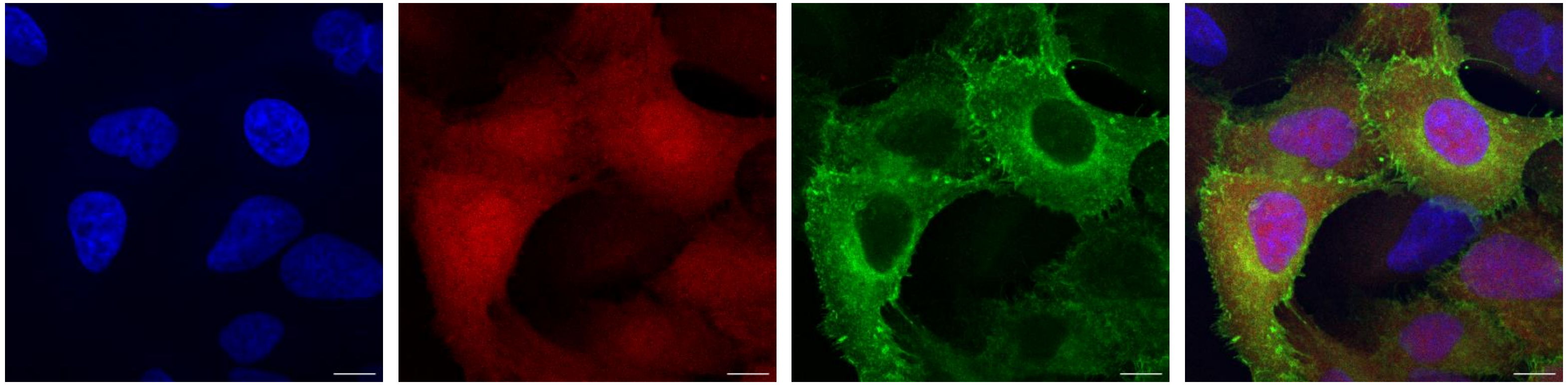

Figure 8E

HA

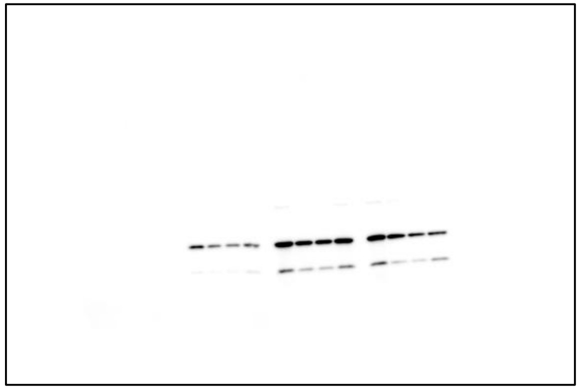

Flag

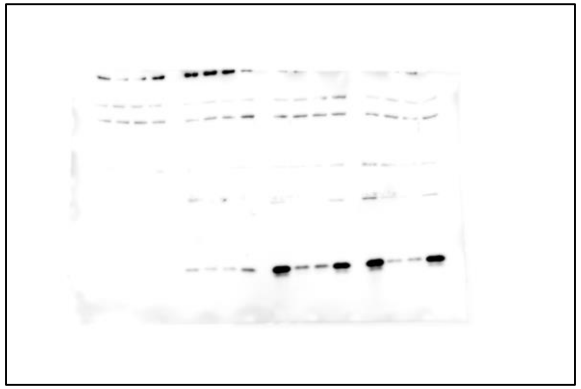

Flag

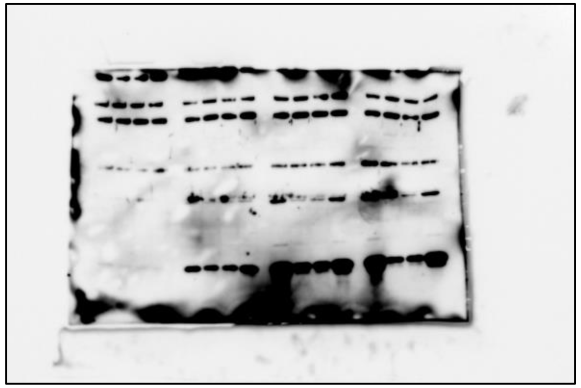

β-actin

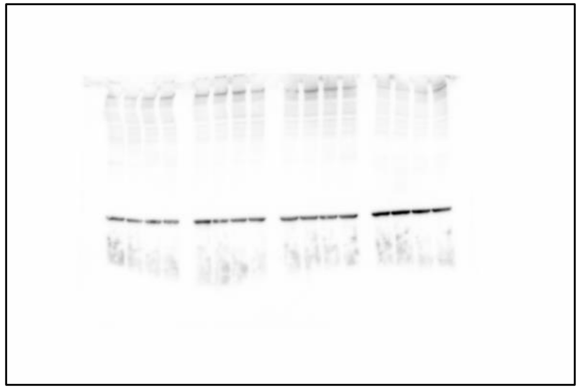

Figure 9

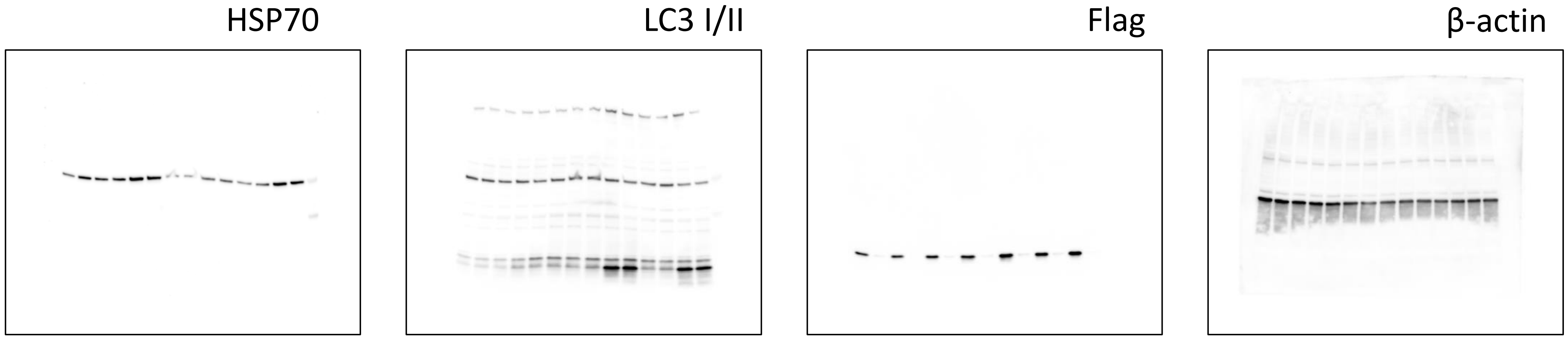

S1A Figure

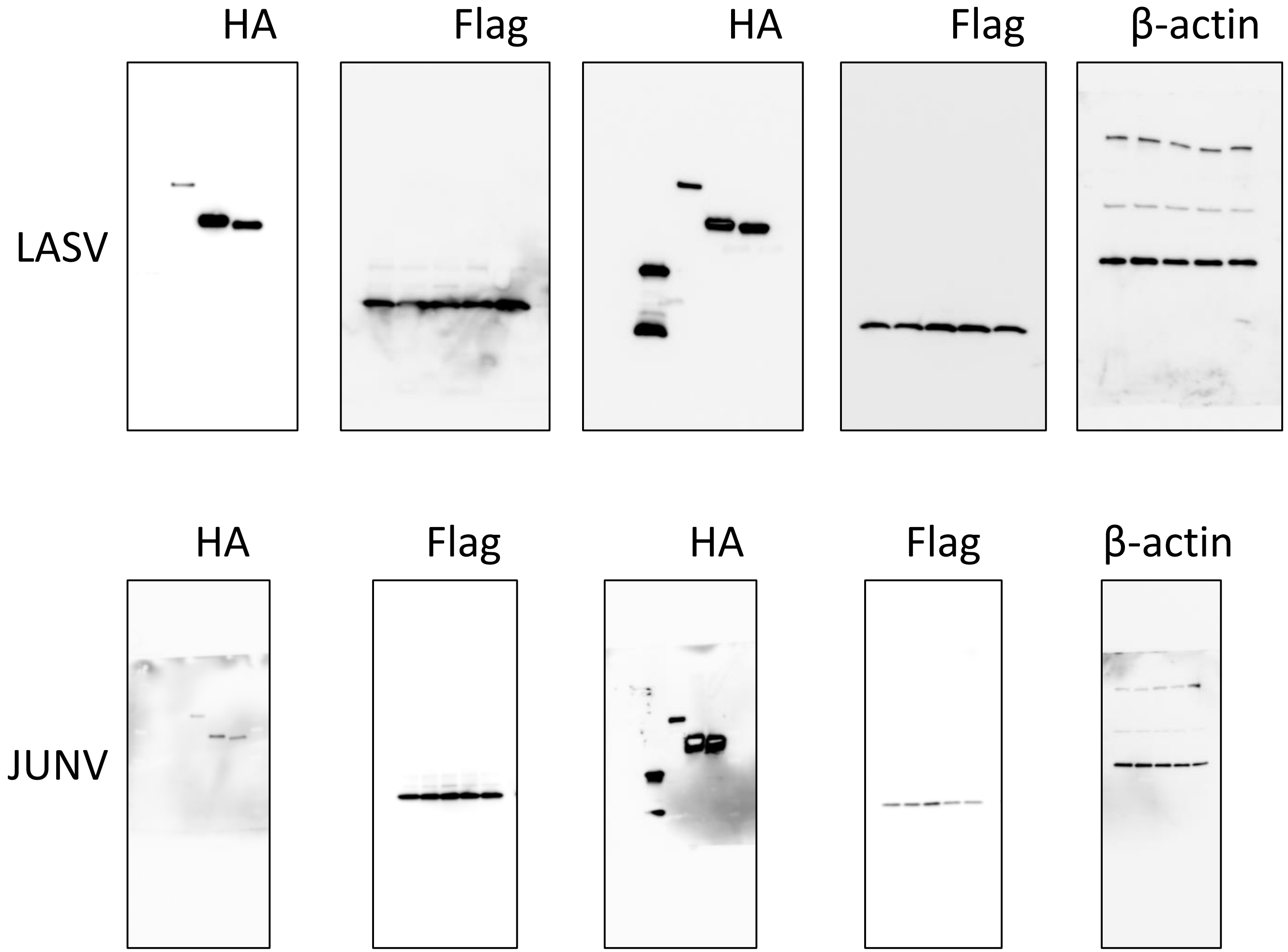

S1C Figure

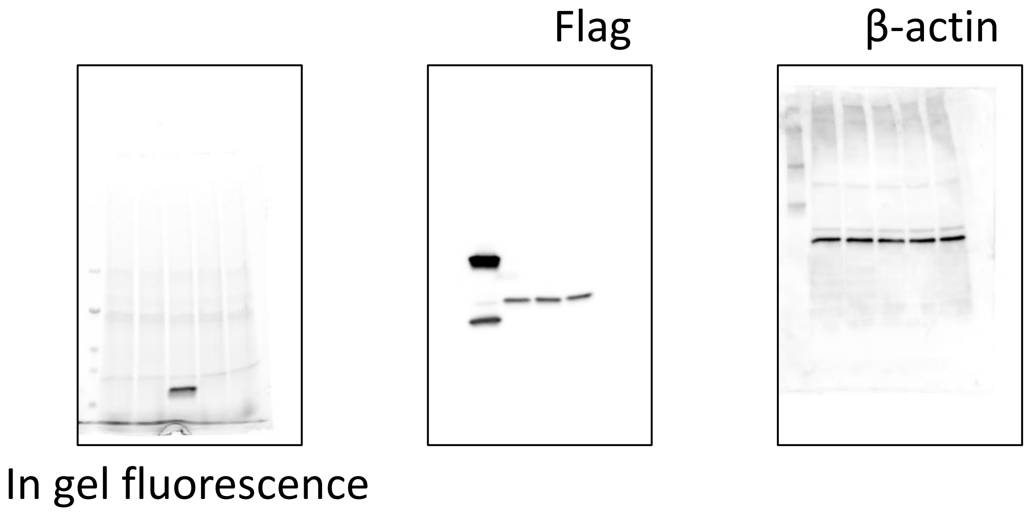

Supplement: S2 Data — (PDF) [file ppat.1012715.s006.pdf]
