# Supplementary material for: Targeting n-myristoyltransferases promotes a pan-Mammarenavirus inhibition through the degradation of the Z matrix protein
Source: PLoS Pathog. 2024 Dec 3;20(12):e1012715. doi: 10.1371/journal.ppat.1012715 (PMC11658702; doi:10.1371/journal.ppat.1012715)
Supplement: Attachment — Submitted filename: Response to reviewers X Carnec et al.docx [file ppat.1012715.s007.docx]

Dear Dr. Baize,

Thank you very much for submitting your manuscript "targeting n-myristoyltransferases promotes a pan-mammarenavirus inhibition through the degradation of the Z matrix protein" for consideration at PLOS Pathogens. As with all papers reviewed by the journal, your manuscript was reviewed by members of the editorial board and by several independent reviewers. In light of the reviews (below this email), we would like to invite the resubmission of a significantly-revised version that takes into account the reviewers' comments.

Thank you for submitting your work for consideration of publication in PLoS Pathogens. Both reviewers think that "the experimental section of the paper has been superbly executed and the results have been clearly presented and nicely illustrated." That being said, there are some additional experiments and controls that still need to be included to improve the overall quality of the work. Please consider their comments, suggestions, and recommendations in both the Summary section and Major/Minor Issue sections carefully when revising the manuscript for a resubmission. Thank you.

We cannot make any decision about publication until we have seen the revised manuscript and your response to the reviewers' comments. Your revised manuscript is also likely to be sent to reviewers for further evaluation.

When you are ready to resubmit, please upload the following:

[1] A letter containing a detailed list of your responses to the review comments and a description of the changes you have made in the manuscript. Please note while forming your response, if your article is accepted, you may have the opportunity to make the peer review history publicly available. The record will include editor decision letters (with reviews) and your responses to reviewer comments. If eligible, we will contact you to opt in or out.

[2] Two versions of the revised manuscript: one with either highlights or tracked changes denoting where the text has been changed; the other a clean version (uploaded as the manuscript file).

Important additional instructions are given below your reviewer comments.

Please prepare and submit your revised manuscript within 60 days. If you anticipate any delay, please let us know the expected resubmission date by replying to this email. Please note that revised manuscripts received after the 60-day due date may require evaluation and peer review similar to newly submitted manuscripts.

Thank you again for your submission. We hope that our editorial process has been constructive so far, and we welcome your feedback at any time. Please don't hesitate to contact us if you have any questions or comments.

Sincerely,

Hinh Ly, Ph.D.
Guest Editor
PLOS Pathogens

Benhur Lee
Section Editor
PLOS Pathogens

Michael Malim
Editor-in-Chief
PLOS Pathogens
orcid.org/0000-0002-7699-2064

***********************

Thank you for submitting your work for consideration of publication in PLoS Pathogens. Both reviewers think that "the experimental section of the paper has been superbly executed and the results have been clearly presented and nicely illustrated." That being said, there are some additional experiments and controls that still need to be included to improve the overall quality of the work. Please consider their comments, suggestions, and recommendations in both the Summary section and Major/Minor Issue sections carefully when revising the manuscript for a resubmission. Thank you.

We thank the Editors @Plos Pathogens and the Reviewers for considering our study and its peer reviewing. All the major and minor issues have been taken into account and we believe that they greatly improved its quality.

The deep(er) reviewing of our manuscript also allowed us to identify misprints and other details that were corrected. We would like to draw your attention with the results related to Fig1. The different parts of the description of this figure were moved compare to the original draft in order to improve the reading. Minor changes were then introduced to connect them.

Reviewer's Responses to Questions

**Part I - Summary**
Please use this section to discuss strengths/weaknesses of study, novelty/significance, general execution and scholarship.

Reviewer #1: The study by Carnec et al demonstrates that arenaviral Z matrix proteins interact with cellular N-Myristoyltransferases 1 and 2 to become myristoylated. Knockout experiments suggest that while both NMT1 and NMT2 can modify the Z protein, the more abundant NMT1 was more biologicially important. This study builds on previous work that observed both Z and SSP myristoylation is important for arenaviral particle abundance and infectivity. Similar to the observations seen when genetically removing the Z myristoylation site, Carnec et al find two NMT inhibitors, IMP1088 and DDD85646, robustly inhibit particle production. They suggest the inhibitor leads to degradation of Z in a proteasome and autophagy independent manner, but this experiment lacks some controls to draw this conclusion. The study is well written and executed well, but a few controls are needed to fully support the conclusions. Overall the study provides additional evidence supporting acylation of arenavirus proteins are critical for their reproduction.

We thank Reviewer #1 for his/her summary; all its major and minor issues drawn to our attention were taken into account.

Reviewer #2: This work by Carnec and colleagues investigates the effect of pharmacological inhibition of host cell N-myristoyltransferases (NMT1 and NMT2) on mammarenavirus replication in cultured cells. Published evidence has shown that the mammarenavirus matrix Z protein and the stable signal peptide (SSP), generated via co- translational processing of the glycoprotein precursor GPC, undergo N-terminal myristoylation by host cell N-myristoyltransferases. Importantly, G2A mutations that prevent myristoylation of Z or SSP have been shown to affect Z mediated virus budding and GP2 mediated fusion activity required to complete the mammarenavirus cell entry process. Although the importance of Z and SSP myristoylation on mammarenavirus multiplication has been documented, these earlier studies were done using pharmacological agents that subsequent have been shown to exert their effects on a rather non-specific and off-target manner. Hence, the significance of the studies by Carnec and colleagues examining the role of N-myristoylation in the mammarenavirus life cycle using the validated on-target NMT1/2 inhibitors DD85646 and IMP1088.

The experimental section of the paper has been superbly executed and the results have been clearly presented and nicely illustrated. The authors should be commended for the extensive and high-quality work done, which supports the main message of the paper, namely that pharmacological inhibition of NMT1 and NMT2 using the validated on-target inhibitors, DD85646 and IMP1088, results in a potent dose-dependent inhibition of mammarenavirus multiplication in cultured cells that is associated with the matrix Z protein being targeted for degradation. These findings represent a significant contribution to our current understanding of mammarenavirus biology and provide also a strong support for investigating the use of NMT1/2 inhibitors as candidate host-directed antivirals to treat infections by human pathogenic mammarenaviruses.

An aspect that needs additional consideration is whether the known role of N-myristoylation in regulation of the host cell innate immunity could have influenced some to the reported findings.

Does treatment with DDD85646 or IMP1088 affects the type 1 interferon response, which could, in turn, affect virus multiplication? Related to this issue, it would be informative to assess whether pharmacological inhibition of NMT1/2 can contribute to PARP and mitochondrial stress mediated antiviral response.

We thank Reviewer #2 for his/her comments.

We measured the IFNβ transcription in A549 cells infected with MOPV treated or not with IMP1088 at 0 h, 24 h and 48 h post infection (S3 Fig). Our results showed that:

i) IMP1088 did not induce IFNβ transcription by itself that could inhibit any further infection,
ii) ii) 48 h post infection, the IFNβ mRNA transcription was highly increased in the presence of IMP1088 compared to the untreated condition,
iii) non-infected cells treated with IMP1088 responded to the type I IFN from infected cells. Our results did not directly support this conclusion but provided a credible explanation for the observed result

Using WCE of the experiments described above, we checked by WB for the presence of full length and cleaved PARP1 forms. Our results showed that neither infection by MOPV nor the treatment by IMP1088 induced an increase in PARP1 expression or its cleavage. Apoptosis does not seem triggered, at least in our experimental conditions. We did not include this “negative” result in the revised version of our manuscript.

It is unclear why the authors have not examined whether treatment with DDD85646 or IMP1088 also results in the expected lack of SSP myristoylation and the expected impact in GP-mediated cell fusion during the virus cell entry process.

We addressed the role of G2A mutation in the revised version of the manuscript (S4 Fig). As expected, the absence of G2 residue in Z did not allowed the rescue of MOPV. One surprising result was the absence of obvious attenuation for the GPC G2A rMOPV compared to its WT counterpart. Our result is in striking contrast with the field, mainly obtained with New World *Mammarenavirus*. As the entry mechanism of New and Old World *Mammarenavirus* is different, it may not be surprising that myristoylation has a different role or significance between these viruses. We also tried to evidence the myristoylation of the SSP of MOPV (or its absence) during infection but we could not gather evidence for it: the detection of the low molecular weight SSP superimposed with the fluorescent reagent of the “click reaction” when assessing the in gel fluorescence.

**Part II – Major Issues: Key Experiments Required for Acceptance**
Please use this section to detail the key new experiments or modifications of existing experiments that should be absolutely required to validate study conclusions.

Generally, there should be no more than 3 such required experiments or major modifications for a "Major Revision" recommendation. If more than 3 experiments are necessary to validate the study conclusions, then you are encouraged to recommend "Reject".

Reviewer #1: Line 209: Figure 3. Please explain how “These results seemed confirmed at the protein level in the matching WCE.” The immunoblots appear similar and based on the methods, different antibodies were used to detect NMT1 and NMT2. Without detailed antibody affinity information can you ever compare the overall expression levels of two different protein on a western? You can claim they are produced similarly across the time scale, but little more.

We agree with Reviewer #1 and modified our conclusions by exchanging “These results seemed confirmed at the protein level in the matching WCE” with “In the matching WB, the expression of NMT1 or NMT2 seemed constant across time and unaffected by infection.”

Why is HA-NMT2 in a separate blot in part B? It makes it difficult to assess relative expression levels.

The HA-NMT2 lane originates from the same membrane/blot as the other lanes presented in Fig 3B but were originally separated from each other’s by 6 lanes. These « cropped » lanes corresponded to various transfected NMT constructs that gave little or no more relevant information to what is shown in the figure. A comment is added to the legend of Fig 3 to inform readers.

In part C, could the “high molecular weight version of NMT2” simply be some low cross reactivity of NMT1 with the NMT2 antibody? How specific are the antibodies? Are the proteins the same size?

NMT1 and NMT2 full length have apparent molecular weight between 55 and 60kDa. The antibodies used to detect NMT1 and NMT2 for the study have been raised against peptides derived from their respective N-terminal regions that are the most divergent ones. Even though we did not detect crossreactivity between the antibodies (Fig 2A), we cannot rule out that the higher molecular band detected on Fig 3C might correspond to NMT1. The text was modified to include this possibility.

Figure 4: I was slightly confused about the inclusion of C75 in the assay. While it clearly did not inhibit the arenaviruses, it also did not alter yellow fever titers. Previous work has suggested that flaviviruses require FASN activity to efficiently replicate. Could C75 simply just not be active? Nothing to be too concerned about with your conclusions, just may want to ensure that C75 is actually inhibiting FASN to include it over a simple DMSO control.
Panel A has so many lines it is somewhat difficult to follow each, can you make the YFV line a different color or dashed lines to help?

FASN is cellular factor involved in the biology of Dengue virus as shown by the report describing its « hijacking » to establish membrane associated replication complexes. The work from Heaton et al (PNAS 2010, PMID: 20855599) primarily showed that Dengue virus is sensitive to C75 treatment in HuH7.5 cells (Fig 1D). The authors also used C75 in BHK cells infected with YFV-17D (Fig 1E) and showed a rather less effective inhibition compared to Dengue virus at concentrations similar to both studies. It is rather surprising that three cellular models were used for three different viruses (ie 1 cell type + 1 virus) and compared to establish the potency of C75 against Flaviviruses. To clarify our results, we removed the C75 and YFV from Fig 4A. YFV is a control for IMP1088 and DDD85646 and modified the text consequently.

We agree with the reviewer for panel A and modified the YFV results with a dashed line representation.

Figure 5. Part A – the three gels are not aligned well. I know the bottom of the gels spread making it tricky, but maybe adding lane markers (1-14) on the top of each one would help. Part B has some missing labels on the right hand side (at least on my copy). I assume the top is an IP and bottom in WCE. Was part B also an anti-FLAP IP? Why does the FLAG IP not more closely match the one in A? Does the addition of the click chem reagents enhance the loss of Z produced or is the effect variable. The HA results are surprising with the amount of FLAG signal seen in the same lanes.

Lane markers were added to the 3 gel parts of Fig 5A to facilitate its understanding and also to Fig 5B to facilitate inter panels comparisons. The corresponding legend to figure 5 was thus modified.

We apologize to the Reviewer for the absence of the missing labels. We certainly made sure that the figures were complete during the submission process.

Fig 5A and 5B present results from Flag-IP and WCE, but detection signals are based on different approaches, fluorescence and chemiluminescent respectively. Because of technical hindrances, we had to run experiments parallel, firstly because we could not quantify the Flag IP eluates as the WCE and therefore used it all for the click reaction, and secondly, after silver staining, we could not transfer protein to PVDF membranes and proceed to WB. We used identically set experiments for Fig 5B except for the addition of the myristate analogue.

We believe that the surprising HA results come from the difference between the concentration of IMP1088 that inhibit the NMT/Z interaction and the *in vitro* effect observed during infection in Fig 4, a fact also mentioned by Reviewer #2. The results obtained in Fig 5 were from transfected HEK293T cells, wherein the expression levels of NMT1 or NMT2 (and Z) are highly increased compared to endogenous ones during infection as in Fig 4. Therefore, in this overexpression conditions, it is not surprising that a higher concentration of IMP1088 is needed to inhibit the interaction between NMTs and its viral substrate, hence explaining these discrepancies.

Figure 9. Is there a control you could probe for to demonstrate some other protein not getting myristoylated is rescued by MG132 (based on your rationale others have identified some). This would control for the drug treatment is working as expected. Currently you have a lack of a result, but no proof that things were working as expected to support your conclusion that it is not a proteasome degradation pathway.

The results related to the use of MG132 and Chloroquine have been taken into account. To evidence the effect of MG132, we detected HSP70 by WB and found that its presence in WCE was increased at 10 µM compare to mock treated cells. A classical detection of LC3-I / LC3-II by WB evidenced the inhibition of the various autophagy pathways also effective at 10 µM. Both catabolic pathways inhibition being demonstrated, our results remained unchanged: we did not observe an increased presence of the Z matrix protein in the WCE.

Through literature roaming, a publication draw our attention regarding the proteasomal activity. The study of Zhang et al (Sc. Advances, 2023. PMID: 38019907) showed that the Rptl2/PSMC1 subunit of the 26S proteasome is myristoylated and its abrogation either by Crispr/Cas9 invalidation or chemical inhibition by IMP1088 lead to the inhibition of what they called membrane associated proteasomes (MAPs). It is therefore likely that part of the proteasomal activity was already inhibited in our experimental conditions. Nevertheless, MAPs do not represent the entire proteasomal activity in cells and the use of MG132 was necessary to fully inhibit it. We added comments related to these findings and ours in the discussion, for what we believe, is an enrichment of it.

Reviewer #2: Despite the excellent technical quality of the present work, there are some technical issues that need additional clarifications and discussion.

1) Levels of NMT1 and NMT2 interacting with Z appear to be rather low based on results shown in Fig. 1B. Moreover, steady state levels of NMT2 appear to be significantly lower than NMT1 levels, which does not seem to be affected by mammarenavirus infection. It is therefore unexpected that levels of NMT1 and NMT2 Co-IP with Z appear to be very similar. Is that possible that NMT2, despite being expressed at lower levels, has a stronger affinity for Z?
We agree with Reviewer #2 regarding the low levels of NMT1 and NMT2 interacting with the Z matrix protein during infection. However, the Reviewer must take into account that as an enzymatic reaction, (with for example a reported k_cat_ around 0.41^s-1^for the GCSVSKKK peptide (Dian et al. 2020, PMID: **32111831**)), the amount of NMT/Z complexes to be detected at any time during infection is likely very limited especially without the use of a crosslinking reactive. Because the Z matrix protein is known to interact with numerous cellular proteins, we did not add such crosslinker into our protocol to further reinforce the specificity and the quality of our results.

Figure 1 aimed to evidence the interaction between the endogenous NMT1 or NMT2 and the Z matrix protein during infection and characterize their main features ectopically (Fig1 and S1). Both enzymes are known to present very conserved active sites. One study solved structure of both enzymes with Myr-coA and the same substrate peptide (Kosciuk et al. 2020, PMID: 32103017 with PDB ID: 6PAU and 6PAV).The authors showed that the kinetics for myristoylation of G2A and K3R Arf6 derived peptides are almost identical for both enzymes (Fig S1C). This result indirectly demonstrated that NMT1 and NMT2 have nearly identical capacities to myristoylate G2 containing N-terminal peptides. It is therefore likely that NMT1 and NMT2 have similar, if not identical, capacities to interact with Z but the N-terminal region of NMT1 and/or the cellular context for the interaction make NMT2 far less used during infection.

2) Western blot results shown in Fig 1D need quantification as levels of Z-FLAG protein appear to be quite different among samples.
The IP and WCE of samples in Fig 1D were originally run on the same gel and probed together with antibodies. Using the same WCE samples as presented in the original figure, we obtained better results for HA, Flag and β-actin and integrated them to the Fig 1D.

3) Results shown in Fig. 5B: it is unclear why levels of Z-FLAG in mock-transfected cells appear to be similarly low to those in transfected cells. It should be also discussed why IMP1088 at 100 nM, a concentration that is effective inhibiting virus multiplication, seems to be unable to prevent the interaction of NMT1 or NMT2 with Z, required for inhibition of Z myristoylation.
The results obtained in Fig 5 were obtained in an ectopic context in another cell line (HEK293T cells) compared to the infection of A549 cells in Fig 4. Therefore, in this overexpression conditions, it is not surprising that a higher concentration of IMP1088 is needed to inhibit the interaction between NMTs and its viral substrate, hence explaining these discrepancies. A comment related to Fig 5 was also added to explain the differences of IMP1088 concentration required to inhibit Z myristoylation between experimental conditions.

4) Results shown in Fig. 4E would be strengthened by conducting a time of addition experiment using NH4Cl treatment to prevent the confounding associated with multiple rounds of infection.

The results of Fig 4E were performed at MOI 0.01 in order to evaluate the capacity of the compounds to inhibit the infection from a small pool of infected cells. Results in Fig 7 and 8 were performed at high MOI in order to minimize the effect of multiple rounds of infection.

Please note that the Y scale name is modified to match with the legend: WT is substituted with mock.

5) Results shown in Fig. 7F seems to conflict with the role of Z as a negative regulator of the activity of the virus ribonucleoprotein complex that is responsible for directing the biosynthetic processes of replication and transcription of the viral genome.

During infection, the treatment of cells with IMP1088 or DDD85646 promotes the post translational degradation of the Z matrix protein. We agree with Reviewer #2 that the absence of Z should unlock the Lpol into an unregulated replication / transcription state of the viral genome. Our results are therefore surprising but we must also emphasize that i) even though, several elegant structural studies demonstrated that Lpol and Z interacted together (the later being capable to inhibit transcription of vRNA), to our knowledge, it has never been observed during infection (for example, Khamina et al. 2017 PMID: 29261807), and ii) albeit mostly degraded, a pool of non myristoylated Z protein, or a part of it might still exist and capable to bind to the Lpol)

Results presented in Fig. 9 do not provide unequivocal evidence supporting the conclusion that Z degradation is a proteasome-independent event. Results from the incorporation of an MG132 dose-response, and a control of a protein known to be targeted for degradation upon N-myristoylation inhibitor can help to clarify this issue.

We thank the Reviewer #2 for his/her comment regarding the proteasome / autophagy independent degradation of the Z matrix protein. Reviewer #1 also addressed this question. Here is a copy of our response also addressed to Reviewer #1:

The results related to the use of MG132 and Chloroquine have been taken into account. To evidence the effect of MG132, we detected HSP70 by WB and found that its presence in WCE was increased at 10 µM compare to mock treated cells. A classical detection of LC3-I / LC3-II by WB evidenced the inhibition of the various autophagy pathways also effective at 10 µM. Both catabolic pathways inhibited, our results remained unchanged: we did not observe an increased presence of the Z matrix protein in the WCE.

Through literature roaming, a publication draw our attention regarding the proteasomal activity. The study of Zhang et al (Sc. Advances, 2023. PMID: 38019907) showed that the Rptl2/PSMC1 subunit of the 26S proteasome is myristoylated and its abrogation either by Crispr/Cas9 invalidation or inhibition by IMP1088 lead to the inhibition of what they called membrane associated proteasomes (MAPs). It is therefore likely that part of the proteasomal activity was already inhibited in our experimental conditions. Nevertheless, MAPs do not represent the entire proteasomal activity in cells and the use of MG132 was necessary to fully inhibit it. We added comments related to these findings and ours in the discussion, for what we believe, is an enrichment of it.

7) Several of the experiments will benefit from the incorporation of the G2A mutant Z protein, which does not undergo N-myristoylation.

Data gathered during our study showed that it was possible to detect interaction between G2A Z and HA-NMT1/2 in ectopic conditions, hence the use of the A5 alanine mutant in Fig 1C. Our results are in line with the two studies identifying that K3 myristoylation was possible (and reversible) when G2 is modified/deleted, demonstrating that an interaction between G2 “less” peptides and NMT1/2 is possible (Dian et al. 2020 and Kosciuk et al 2020). Therefore, the absence of the N-terminal G residue does not prevent interaction with NMTs but myristoylation.

The S1C Fig provided evidence that the Z matrix protein of MOPV is neither G2 nor K3 myristoylated when the G2A mutation is introduced in the sequence of MOPV Z protein. Reverse genetics rescue of G2A Z mutants were not viable and reinforced our results (see supplemental S4 Figure).

8) The authors should comment on published work defining the interactome of JUNV Z protein where a large number of Z-host cell interacting proteins, but not NMT1/2 , were identified.

We added comments related to the Z interactome in the discussion. It is not surprising that the NMT/Z interaction was not detected because of its enzymatic nature. When the cells are lysed, this “snapshot” only allows the recovery of the Z being myristoylated, while other more stable and long lasting interactions are likely easier to collect and detect.

**Part III – Minor Issues: Editorial and Data Presentation Modifications**
Please use this section for editorial suggestions as well as relatively minor modifications of existing data that would enhance clarity.

Reviewer #1: Line 29: Change “If” to “While”

Changed

Line 104: Change “or” to “and”

Changed

Figure 1 A – the FLAG IP immunoblot and the WCE in gel fluorescence could be aligned and cropped better. The Z band is touching the edge of the crop in the top and in the bottom, the gel is misaligned and the wt lane is half missing.

The original picture was resized, realigned and incorporated into the figure.

Figure 1 B – The Flag immunoblot appears overly saturate (burnt bands) is there a shorted exposure? Similarly, although not quite as bad, the NMT2 blot for WCE?

The WB of Fig 1B show Flag IP and WCE samples run parallel on the same membrane (one membrane for NMT1 detection, another one for NMT2 detection). The pictures were analyzed with ImageJ with settings allowing a good visualization of the co-immunoprecipitated NMT1 and NMT2; unfortunately with a saturation signal for Flag and HA detection in IP and WCE. We choose to keep these settings for WB visualization of the IP and WCE. We believe that changing the WB picture of IP and WCE for a shorter exposure/saturation or different settings with ImageJ may mislead the interpretations: only a small proportion of the endogenous NMTs interacts with Z, reflecting the enzymatic nature of this interaction (and its difficulty to detect). By decreasing the band intensity in IP/WCE, one could conclude otherwise.

Figure S1 part C and Line 227, 231, 353, 698 remove the “e” at the end of actin

All “actine” were changed for actin.

Line 205: Would be helpful to add citations here again

We added references related to the NMT1 dependency for HIV-1 and Picornaviruses and modified the text accordingly.

Line 345: The test say rMOPVmCherryHA but the figure says mCherryFLAG. Were these cells actually stained or just monitored for mCherry in flow?

We thank Reviewer #2 for its attention. The mCherryHA was changed for mCherryFlag. To make sure that detection by Facs was even between the Z-Flag and the mCherryFlag, both were stained with an AF647 conjugated anti-Flag antibody.

Figure 7 B: May want to alter the Axes to “% of flag or HA” or “ % of tag +” rather than (Flag/HA) I was confused and thought you were showing a ratio of FLAG/HA positive
The axe legend of Fig 7B was changed for “ MFI of flag or ha positive cells (% to Mock)

Line 530: Wasn’t this data from HAP1 cells, not A549?

HAP1 NMT1_ko_ evidenced the dependency of Mammarenavirus to this enzyme. We tested the role of NMT1 and NMT2 in A549 cells during infection using siRNA mediated knockdown and obtained similar results as in HAP1 cells. Because we did not include this result in the manuscript, we modified the text of the discussion accordingly.

Reviewer #2: The paper is in overall well written, but the introduction section would benefit from some additional editing to eliminate unclear sentences like “Mammarenavirus genus family have a worldwide...”, and to clarify that LASV, JUNV and other hemorrhagic fever causing mammarenaviruses do not cause epidemics per se, but rather they are endogenous to specific geographic locations.
The introduction was clarified as suggested by the Reviewer.

The authors should comment on the discussion section on the small molecule NMT inhibitor PCLX-001, which has been shown to be safe and well tolerated in humans, supporting the interest of exploring the repurposing of NMT inhibitors to treat infections by human pathogenic mammarenaviruses.

Comments related to PCLX-001 were added in the discussion.

I would suggest to incorporate a brief comment in the M&M section of the paper stating that experiments with LASV, LUJV, JUNV and MACV have been done in appropriate BSL4 containment facilities.

The comment related to the BSL4 experimental work was moved from the acknowledgment to the M&M section of the publication.

PLOS authors have the option to publish the peer review history of their article ([what does this mean?](https://urldefense.com/v3/__https:/c05y1x9s.r.us-east-2.awstrack.me/L0/https:*2F*2Fjournals.plos.org*2Fplospathogens*2Fs*2Feditorial-and-peer-review-process*23loc-peer-review-history/1/010f01918f97db6f-b879e380-3fa6-4f80-8a60-5c475ce802f3-000000/DH_2P6_2-tZ4Frq1pdt2S3WlqkU=173__;JSUlJSUl!!JFdNOqOXpB6UZW0!p102co-Pl1rpxFbjtjqKTxbc9LIT0zceMZXAZRF-CgaxPyAgcFKcn4c6cMkliU7KSRRmjzpuV0kgD9q_mZlh-BE$)). If published, this will include your full peer review and any attached files.


If you choose “no”, your identity will remain anonymous but your review may still be made public.


**Do you want your identity to be public for this peer review?** For information about this choice, including consent withdrawal, please see our [Privacy Policy](https://urldefense.com/v3/__https:/c05y1x9s.r.us-east-2.awstrack.me/L0/https:*2F*2Fwww.plos.org*2Fprivacy-policy/1/010f01918f97db6f-b879e380-3fa6-4f80-8a60-5c475ce802f3-000000/4zerYr3ixfh5P4t86BllQ3yOjhY=173__;JSUl!!JFdNOqOXpB6UZW0!p102co-Pl1rpxFbjtjqKTxbc9LIT0zceMZXAZRF-CgaxPyAgcFKcn4c6cMkliU7KSRRmjzpuV0kgD9q_l9vl2AU$).

Reviewer #1: No

Reviewer #2: No

Figure Files:

While revising your submission, please upload your figure files to the Preflight Analysis and Conversion Engine (PACE) digital diagnostic tool, [https://pacev2.apexcovantage.com](https://urldefense.com/v3/__https:/c05y1x9s.r.us-east-2.awstrack.me/L0/https:*2F*2Fpacev2.apexcovantage.com*2F/1/010f01918f97db6f-b879e380-3fa6-4f80-8a60-5c475ce802f3-000000/BIHSU4N-t2FPkfMe9eQUPSZW98w=173__;JSUl!!JFdNOqOXpB6UZW0!p102co-Pl1rpxFbjtjqKTxbc9LIT0zceMZXAZRF-CgaxPyAgcFKcn4c6cMkliU7KSRRmjzpuV0kgD9q_-qS8RV0$). PACE helps ensure that figures meet PLOS requirements. To use PACE, you must first register as a user. Then, login and navigate to the UPLOAD tab, where you will find detailed instructions on how to use the tool. If you encounter any issues or have any questions when using PACE, please email us at [figures@plos.org](mailto:figures@plos.org).

Data Requirements:

Please note that, as a condition of publication, PLOS' data policy requires that you make available all data used to draw the conclusions outlined in your manuscript. Data must be deposited in an appropriate repository, included within the body of the manuscript, or uploaded as supporting information. This includes all numerical values that were used to generate graphs, histograms etc..For an example see here on PLOS Biology: [http://www.plosbiology.org/article/info%3Adoi%2F10.1371%2Fjournal.pbio.1001908#s5](https://urldefense.com/v3/__http:/www.plosbiology.org/article/info*3Adoi*2F10.1371*2Fjournal.pbio.1001908*s5__;JSUlIw!!JFdNOqOXpB6UZW0!p102co-Pl1rpxFbjtjqKTxbc9LIT0zceMZXAZRF-CgaxPyAgcFKcn4c6cMkliU7KSRRmjzpuV0kgD9q_GgCRKjw$).

Reproducibility:

To enhance the reproducibility of your results, we recommend that you deposit your laboratory protocols in protocols.io, where a protocol can be assigned its own identifier (DOI) such that it can be cited independently in the future. Additionally, PLOS ONE offers an option to publish peer-reviewed clinical study protocols. Read more information on sharing protocols at [https://plos.org/protocols?utm_medium=editorial-email&utm_source=authorletters&utm_campaign=protocols](https://urldefense.com/v3/__https:/c05y1x9s.r.us-east-2.awstrack.me/L0/https:*2F*2Fplos.org*2Fprotocols*3Futm_medium=editorial-email*26utm_source=authorletters*26utm_campaign=protocols/1/010f01918f97db6f-b879e380-3fa6-4f80-8a60-5c475ce802f3-000000/2K2mVvUeQvQqOfa3Lp5uFgq-DGI=173__;JSUlJSUl!!JFdNOqOXpB6UZW0!p102co-Pl1rpxFbjtjqKTxbc9LIT0zceMZXAZRF-CgaxPyAgcFKcn4c6cMkliU7KSRRmjzpuV0kgD9q_xMb9H9E$)
